# Automated quantification of T1 and T2 relaxation times in liver mpMRI using deep learning: a sequence-adaptive approach

**DOI:** 10.1186/s41747-025-00596-9

**Published:** 2025-06-14

**Authors:** Lukas Zbinden, Samuel Erb, Damiano Catucci, Lars Doorenbos, Leona Hulbert, Annalisa Berzigotti, Michael Brönimann, Lukas Ebner, Andreas Christe, Verena Carola Obmann, Raphael Sznitman, Adrian Thomas Huber

**Affiliations:** 1 ARTORG Center for Biomedical Engineering Research, University of Bern, Bern, Switzerland; 2 Department of Diagnostic, Interventional and Pediatric Radiology, Inselspital, Bern University Hospital, Bern, Switzerland; 3 Graduate School for Health Sciences, University of Bern, Bern, Switzerland; 4 Hepatology, Department of Visceral Surgery and Medicine, Inselspital, Bern University Hospital, Bern, Switzerland; 5 Department of Radiology and Nuclear Medicine, Luzerner Kantonsspital, University Teaching and Research Hospital, University of Lucerne, Lucerne, Switzerland; 6 Department of Radiology, Beau-Site, Hirslanden, Bern, Switzerland

**Keywords:** Chronic liver disease, Deep learning, Liver phenotyping, Multiparametric MRI, Quantification

## Abstract

**Objectives:**

To evaluate a deep learning sequence-adaptive liver multiparametric MRI (mpMRI) assessment with validation in different populations using total and segmental T1 and T2 relaxation time maps.

**Methods:**

A neural network was trained to label liver segmental parenchyma and its vessels on noncontrast T1-weighted gradient-echo Dixon in-phase acquisitions on 200 liver mpMRI examinations. Then, 120 unseen liver mpMRI examinations of patients with primary sclerosing cholangitis or healthy controls were assessed by coregistering the labels to noncontrast and contrast-enhanced T1 and T2 relaxation time maps for optimization and internal testing. The algorithm was externally tested in a segmental and total liver analysis of previously unseen 65 patients with biopsy-proven liver fibrosis and 25 healthy volunteers. Measured relaxation times were compared to manual measurements using intraclass correlation coefficient (ICC) and Wilcoxon test.

**Results:**

Comparison of manual and deep learning-generated segmental areas on different T1 and T2 maps was excellent for segmental (ICC = 0.95 ± 0.1; *p* < 0.001) and total liver assessment (0.97 ± 0.02, *p* < 0.001). The resulting median of the differences between automated and manual measurements among all testing populations and liver segments was 1.8 ms for noncontrast T1 (median 835 *versus* 842 ms), 2.0 ms for contrast-enhanced T1 (median 518 *versus* 519 ms), and 0.3 ms for T2 (median 37 *versus* 37 ms).

**Conclusion:**

Automated quantification of liver mpMRI is highly effective across different patient populations, offering excellent reliability for total and segmental T1 and T2 maps. Its scalable, sequence-adaptive design could foster comprehensive clinical decision-making.

**Relevance statement:**

The proposed automated, sequence-adaptive algorithm for total and segmental analysis of liver mpMRI may be co-registered to any combination of parametric sequences, enabling comprehensive quantitative analysis of liver mpMRI without sequence-specific training.

**Key Points:**

- A deep learning-based algorithm automatically quantified segmental T1 and T2 relaxation times in liver mpMRI.
- The two-step approach of segmentation and co-registration allowed to assess arbitrary sequences.
- The algorithm demonstrated high reliability with manual reader quantification.
- No additional sequence-specific training is required to assess other parametric sequences.
- The DL algorithm has the potential to enhance individual liver phenotyping.

**Graphical Abstract:**

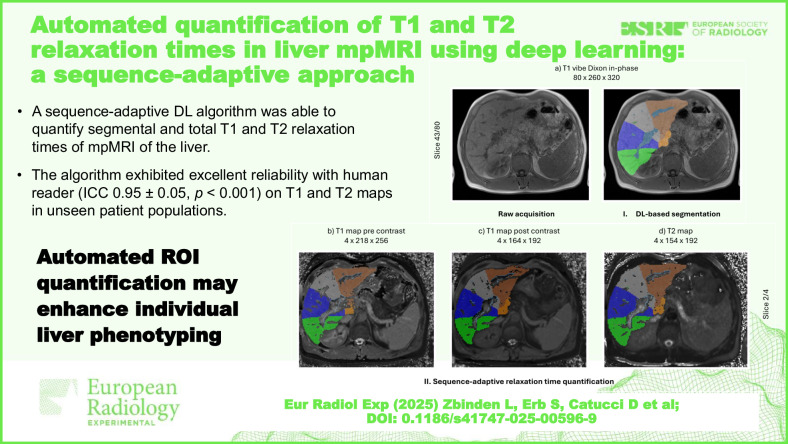

## Background

Multiparametric magnetic resonance imaging (mpMRI) has emerged as a pivotal non-invasive imaging technology for detecting, characterizing, and monitoring chronic liver disease (CLD) [1–6]. For the quantitative assessment of liver segmental parenchyma, mpMRI integrates various parametric sequences, including T1 and T2 mapping [7, 8], proton density fat fraction (PDFF) [9–11], diffusion-weighted intra-voxel incoherent motion (IVIM) [12], and magnetic resonance elastography [13–15]. In liver fibrosis, T1 relaxation time is increased before and decreased after contrast medium administration, while T2 relaxation time is increased in inflammation but not in fibrosis [16]. These data may be combined with PDFF fat quantification, magnetic resonance elastography liver stiffness measurement, and IVIM microperfusion analysis. Thus, mpMRI may enhance the accuracy of diagnosis and pave the way for state-of-the-art liver phenotyping, decision-making and personalized treatment planning.

However, manual quantification of mpMRI data is time-consuming, multiplied by the number of measured locations in different liver segments, and by the number of different sequences. To facilitate routine analysis of combined multiple liver parameters in different liver segments, an automated analysis tool is needed. Such an approach would allow to quantify combined multiparametric information in different liver segments across time, facilitating non-invasive diagnosis, clinical decision-making, and monitoring. Beyond those obvious clinical implications, quantification of mpMRI data is instrumental in research, contributing to the development of novel non-invasive diagnostic techniques in liver imaging and hepatology.

An automatic and sequence-adaptive procedure for mpMRI quantification could alleviate this manual but critical burden on radiologists. Sequence-adaptiveness allows the procedure to dynamically and automatically adapt its computations to any mpMRI sequence as they become available. Deep learning (DL)-based image analysis techniques may provide automated, fast, and reliable results [17], but require separate algorithm training for each new mpMRI sequence and different disease conditions [18]. Therefore, algorithms have been developed that work only on T1 or T2 maps in the heart [19, 20] or combine limited mpMRI data for risk stratification in a specific clinical entity such as endometrial cancer [21]. To our knowledge, there is currently no algorithm that integrates and combines “sequence-adaptive” analysis of previously unseen segmental mpMRI data of the liver.

We hypothesized that a two-step procedure involving a trained algorithm for liver segmental parenchyma and liver vessels on standard 3-mm thickness noncontrast T1 “volumetric interpolated breath-hold examination” (VIBE) Dixon in-phase images, along with co-registration to previously unseen mpMRI data using physical coordinate space affine transformations, would allow to solve this issue. The purpose of this study was to develop an automated, mpMRI sequence-adaptive algorithm and evaluate its performance in unseen segmental and total liver T1 and T2 relaxation time mapping quantification, both before and after contrast medium administration.

## Methods

### Study participants

This was a retrospective analysis of prospectively acquired data to train and validate a new DL-based algorithm. The local Ethics Committee (Bern cantonal ethics committee, Bern, Switzerland, #2017-01695, 01/2018) approved the study, which adhered to the Declaration of Helsinki. All patients gave written informed consent to participate in the study. Patient deidentification occurred during sequence conversion from Digital Imaging and Communications in Medicine (DICOM) to Neuroimaging Informatics Technology Initiative (NIfTI-1) format. The authors had full access to and take full responsibility for the integrity of the data.

### Datasets

**Fig. 1:**
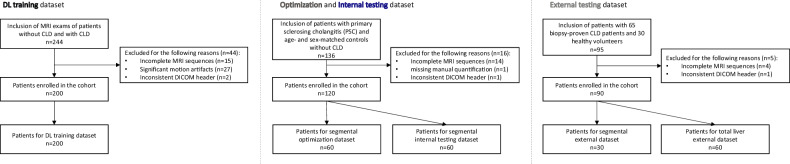
Dataset curation and inclusion flowchart

Five distinct datasets were curated from three study cohorts (Fig. 1). The DL training dataset consisted of 200 liver MRI without focal liver lesions or prior surgery, including a noncontrast 3-mm thickness T1-weighted VIBE Dixon in-phase sequence, as previously reported [22]. This prior article dealt with volumetric analysis of the liver segments and their vessels, whereas this study built on those segmentation results for sequence-adaptive co-registration with different parametric T1 and T2 relaxation time maps. The optimization and internal testing dataset consisted of 136 multiparametric liver MRI from patients with primary sclerosing cholangitis and age- and sex-matched controls without CLD.

An external testing dataset was included, consisting of 94 multiparametric liver MRI, with 65 biopsy-proven CLD patients and 30 healthy volunteers. After excluding patients with incomplete MRI sequences (*n* = 42), inconsistent DICOM header (*n* = 4), the final datasets were: training (*n* = 200), optimization and internal testing (*n* = 120, 58 patients with primary sclerosing cholangitis and 62 patients without CLD), and external testing (*n* = 90). The optimization and internal testing dataset was split equally into optimization (*n* = 60) and internal testing (*n* = 60). The external testing dataset was divided into total liver external dataset (*n* = 60; 35 CLD patients and 25 volunteers) and segmental external dataset (*n* = 30 CLD patients). The following patients were excluded from all datasets: those less than 18 years of age; those denying consent; and those with prior focal liver lesions greater than 2 cm, prior liver resection or interventions; as well as patients with cholestatic liver disease. The sizes of the optimization and testing datasets reflect the availability of imaging data that met the inclusion criteria. While smaller, these datasets were selected to provide diverse coverage of clinical cases relevant to validate the DL algorithm’s robustness and generalizability.

### Magnetic resonance imaging

All datasets consisted of a noncontrast 3 mm T1 VIBE Dixon in-phase acquisition, while the optimization and testing datasets (datasets 2, 3, and 4) additionally included parametric relaxation time mapping sequences at four levels over the liver with 20 mm to 32 mm distance between slices: a noncontrast 8-mm thickness modified Look-Locker (MOLLI) T1 map, an 8-mm thickness multiecho fast spin-echo T2 map, and a post-contrast 8-mm thickness modified Look-Locker (MOLLI) T1 map after 15 min. The MRI parameters of the five datasets used in this study are summarized in Table 1.

**Table 1:** MRI parameters

Characteristic	Dataset																
	Training (*n* = 200)	Optimization, segmental (*n* = 60)				Internal testing, segmental (*n* = 60)				External testing, segmental (*n* = 30)				External testing, total liver (*n* = 60)			
Scanner model	Siemens Magnetom Prisma^fit^, Area	Siemens Magnetom Prisma^fit^, Area, Skyra				Siemens Magnetom Area, Skyra				Siemens Magnetom Prisma^fit^				Siemens Magnetom Prisma^fit^			
Field strength (T)	1.5, 3.0	1.5, 3.0				1.5, 3.0				3.0				3.0			
Sequence	T1 vibe Dixon	T1 vibe Dixon	T1 map pre-contrast	T1 map post-contrast	T2 map	T1 vibe Dixon	T1 map pre-contrast	T1 map post-contrast	T2 map	T1 vibe Dixon	T1 map pre-contrast	T1 map post-contrast	T2 map	T1 vibe Dixon	T1 map pre-contrast	T1 map post-contrast	T2 map
Pixel spacing (mm^2^)	0.94 × 0.94 to 1.56 × 1.56	1.19 × 1.19, 1.25 × 1.25, 1.31 × 1.31	1.41 × 1.41, 1.48 × 1.48, 1.56 × 1.56, 1.88 × 1.88	1.41 × 1.41, 1.48 × 1.48, 1.56 × 1.56, 1.88 × 1.88, 2.08 × 2.08	1.88 × 1.88, 1.98 × 1.98, 2.08 × 2.08	1.19 × 1.19, 1.25 × 1.25, 1.31 × 1.31	1.41 × 1.41, 1.48 × 1.48, 1.56 × 1.56	1.41 × 1.41, 1.88 × 1.88, 1.98 × 1.98, 2.08 × 2.08	1.88 × 1.88, 1.98 × 1.98, 2.08 × 2.08	1.19 × 1.19	1.41 × 1.41, 1.48 × 1.48	1.41 × 1.41, 1.48 × 1.48	1.88 × 1.88, 1.98 × 1.98	1.19 × 1.19, 1.25 × 1.25	1.41 × 1.41, 1.48 × 1.48, 1.56 × 1.56	1.41 × 1.41, 1.48 × 1.48, 1.56 × 1.56	1.88 × 1.88, 1.98 × 1.98, 2.08 × 2.08, 2.19 × 2.19
Slice thickness (mm)	3	3	8	8	8	3	8	8	8	3	8	8	8	3	8	8	8
Spacing between slices (mm)	3	3	32	32	32	3	32	32	32	3	28, 32	28, 32	28, 32	3	20, 24, 25.6, 28, 32	20, 24, 25.6, 28, 32	20, 24, 25.6, 28, 32
Axial dimensions (pixel)	(64, 200, 320) to (104, 210, 320) to (64, 270, 320)	(64, 260, 320), (72, 240, 320), (80, 260, 320), (88, 260, 320), (96, 260, 320)	(4, 226, 256), (4, 154, 192), (4, 218, 256)	(4, 218, 256), (4, 164, 192), (4, 170, 192), (4, 160, 192)	(4, 154, 192), (4, 160, 192), (4, 170, 192)	(72, 260, 320), (80, 260, 320), (88, 260, 320)	(4, 218, 256)	(4, 164, 192), (4, 218, 256)	(4, 152, 192), (4, 154, 192), (4, 162, 192)	(96, 260, 320), (80, 260, 320), (72, 260, 320)	(4, 218, 256)	(4, 218, 256)	(4, 154, 192)	(72, 260, 320), (96, 260, 320), (64, 260, 320), (96, 250, 320), (72, 240, 320), (80, 260, 320)	(4, 218, 256)	(4, 218, 256)	(4, 154, 192)
Intensity ranges	0–3,425	0–2,028	0–4,095	0–4,095	0–409.5	0–1,924	0–4,095	0–4,095	0–409.5	0–1,895	0–4,095	0–4,095	0–409.5	0–1,377	0–4,095	0–4,095	0–409.5

Pixel spacing values were rounded to 2 decimal places

*MR* Magnetic resonance

### Preprocessing and manual mapping quantification

Liver segmental, portal vein and hepatic vein contours were manually annotated slice-by-slice on 3-mm thickness T1-weighted VIBE Dixon in-phase images for the entire training dataset (*n* = 200) by trained radiology residents with 4 (D.C.) or 1 (L.H.) years of experience in liver, and corrected by an abdominal radiologist with 10 years’ experience in liver imaging (M.B.) using ITK-SNAP [23] (version 3.8.0). On the optimization and testing datasets, segmental and whole liver relaxation times were manually determined by drawing regions of interest (ROI) in every liver segment by two trained residents with 3 (S.E.) or 4 (D.C.) years of experience in liver MRI, and reviewed by a second reader with 8 years of experience in liver MRI (J.B.K.), using a dedicated picture archiving and communication system (PACS) (Sectra IDS7, version 21.2, Sectra AB). All polygonal ROIs, including liver parenchyma of Couinaud segments I to VIII, were carefully delineated on the four axial slices of the T1 and T2 mapping sequences, excluding liver vessels (hepatic and portal veins). For segments appearing on two or more consecutive slices, ROIs were drawn on all slices and averaged, weighted by the ROI size.

### Deep learning algorithm

**Fig. 2:**
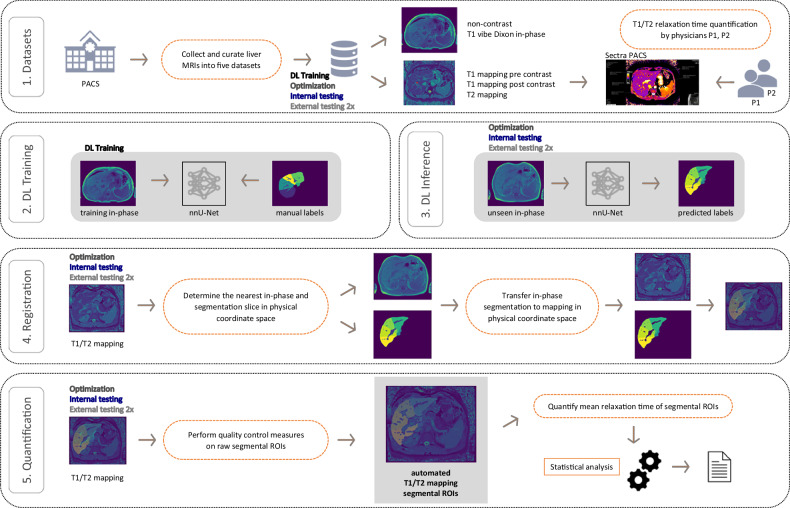
Study workflow of automated liver MRI T1 and T2 mapping quantification. 1. MRI noncontrast Dixon in-phase and T1 / T2 mapping sequences were retrieved from the hospital’s picture archiving and communication system (PACS) and manually quantified for segmental relaxation times. 2. Liver segmental contours and liver veins were manually labeled slice-by-slice (dataset *n* = 200) to train the nnU-Net algorithm. 3. The trained nnU-Net was then used for automated liver segmental labeling, excluding liver portal and hepatic veins, on datasets that were previously unseen by the DL algorithm, including an optimization dataset (*n* = 60), an internal testing dataset (*n* = 60), and an external testing dataset (*n* = 30 for segmental analysis and *n* = 60 for total liver analysis). 4. The generated liver segmental labels were co-registered to parametric T1 and T2 mapping sequences acquired simultaneously in the same physical coordinate space. 5. Finally, the resulting segmental regions of interest (ROIs) on the parametric maps were post-processed for quality control. Parametric relaxation times were then quantitatively and statistically compared to manual direct quantifications on the parametric maps

The workflow of our study with a novel two-step approach of segmentation and co-registration is illustrated in Fig. 2. In a first step, the nnU-Net segmentation algorithm [24] was trained on the training dataset, following the approach described in [22], to delineate liver segments and vessels according to the Couinaud classification system [25]. The model assigned each voxel to one of twelve categories: nine liver segments (dividing the liver into functionally independent units I–VIII with segment IV split into IVa and IVb), portal vessels, hepatic vessels, or background (representing areas outside the liver). Fivefold cross-validation was utilized for neural network training, with each fold comprising 160 liver MRI for training and 40 for validation, on the noncontrast 3 mm thickness T1-weighted VIBE Dixon in-phase sequence dataset. The training procedure and nnU-Net architecture adhered to those published previously [22, 24]. We used a loss function combining Dice loss and cross-entropy loss, the Adam optimizer with an initial learning rate of 3 × 10^−^⁴, and a learning rate scheduler reducing the rate to a minimum of 10^−^⁶ based on training and validation loss averages. Data augmentation during training included random rotations, scaling, elastic deformations, gamma correction, and mirroring. The nnU-Net architecture remained unchanged. To be compatible with the neural network input file format and the proposed algorithm, all MRI sequences in native Digital Imaging and Communications in Medicine (DICOM) format were converted to the Neuroimaging Informatics Technology Initiative (NIfTI-1) format. The following Python libraries were used for data conversion and curation: opencv-python 4.6.0.66, scikit-image 0.17.2, dicom2nifti 2.4.6, SimpleITK 2.0.2, NiBabel 3.2.1. No other preprocessing was applied to the mapping sequences. Network optimization ran for 150 epochs with a batch size of two, utilizing a NVIDIA GeForce RTX 3090 GPU.

### Affine co-registration and quality control measures

In a second step, the trained segmentation network labeled unseen datasets from the optimization and testing datasets. Labeled liver segments were co-registered to raw noncontrast and contrast-enhanced T1 and T2 relaxation maps, which were acquired during the same MRI session, by voxel-wise affine transformations in the shared physical coordinate space of the MRI scanner. For more robust quantification on the relaxation time maps, additional configurable algorithmic components were devised as quality control measures for the transformed liver labels. ROI erosion: removes potentially ambiguous voxels at the ROI boundary; minimum ROI size per slice: if the size of a ROI is below a threshold, the algorithm omits the quantification; removal of voxel outliers: makes the ROI quantification more robust against image artifacts; high entropy detection: if the amount of spatial entropy (indicating areas of poor data quality including noise and image artifacts) inside a ROI is too high, the algorithm omits the quantification; source slice subselection: the stringently obtained T1-weighted VIBE Dixon in-phase slice might not be correct in the case of patient breathing, thus select a neighboring slice with a higher normalized cross-correlation inside the ROI. Technical details of the components are in Appendix E1 (Supplementary Material). The algorithm always calculated the ROI mean voxel value volumetrically from all axial slices containing the same ROI.

### Optimization and validation

The most effective algorithm configuration was found through hyperparameter optimization using a grid search on the optimization dataset. Hyperparameters were chosen as follows: ROI erosion was set to none, small, or large to evaluate the impact of boundary smoothing; minimum ROI size thresholds of 50, 75, or 100 voxels ensured exclusion of insignificant regions; voxel outliers were removed using the total liver median ± 3 * median absolute deviation to handle extreme intensity variations; high entropy detection was applied in four variants to optimize the balance between detection sensitivity and specificity; and source slice subselection used neighborhood sizes of two or three slices to address breathing artifacts. The algorithm was optimized using a custom objective function to balance maximizing the mean intraclass correlation coefficient (ICC), minimizing ICC variability, and increasing the number of ROI measurements. Technical details are in Appendix E2 (Supplementary Material). The optimized sequence-adaptive mpMRI quantification algorithm was subsequently validated on unseen internal and external testing datasets for total and segmental analysis of noncontrast and post-contrast T1 and T2 relaxation times.

### Statistical analysis

Dataset characteristics were compared using a Kruskal–Wallis test for continuous variables and a χ^2^ test for categorical variables. Quantification reliability was compared using a one-way random, single-measure intraclass correlation coefficient analysis ICC (1, 1) for segmental and total liver analysis, focusing on absolute agreement [26]. An ICC value below 0.5 indicates poor reliability, between 0.5 and 0.75 moderate reliability, between 0.75 and 0.9 good reliability, and any value above 0.9 indicates excellent reliability [27]. Concordance was evaluated with Deming regression analysis [28] and Spearman’s rho ($\rho$). T1 and T2 relaxation times were compared using a paired Wilcoxon test, and 95% confidence intervals were calculated using bootstrap percentiles. Statistical significance level was set at α = 0.05. All analyses were conducted using Python (Python 3.6.13, SciPy 1.5.4, scikit-learn 0.24.2 Matplotlib 3.3.3, Pingouin 0.3.12).

## Results

### Dataset characteristics

Baseline clinical characteristics of the datasets are shown in Table 2. The optimization and internal testing datasets consisted of primary sclerosing cholangitis patients and age- and sex-matched controls, with median ages of 50 and 51 years, and male proportions of 63 and 62%, respectively. The external testing datasets included patients with biopsy-proven CLD and healthy volunteers, with median ages of 54 and 45 years for the segmental and total liver datasets, respectively, and male proportions of 50 and 52%. The liver volumes varied significantly both within and between datasets, with a mean ± standard deviation of 1,026 ± 632 cm^3^ in the optimization dataset and 1,723 ± 429 cm^3^ in the segmental external testing dataset, reflecting the intended heterogeneity. All datasets for training, optimization and testing included both patients with and without CLD to increase the robustness of the analysis. All datasets contained identical standard noncontrast T1 VIBE Dixon 3 mm acquisitions of the liver, while datasets 2–5 additionally contained noncontrast and contrast-enhanced T1 relaxation time maps, as well as T2 relaxation maps. These parametric maps were obtained in different patient populations and had different pixel spacings and slice thicknesses compared to the T1-weighted VIBE Dixon acquisitions.

**Table 2:** Characterization of the five study datasets

	Training (*n* = 200)	Optimization, segmental (*n* = 60)	Internal testing, segmental (*n* = 60)	External testing, segmental (*n* = 30)	External testing, total liver (*n* = 60)	*p*-value
Age (years)	52 (38–62)	50 (40–59)	51 (37–63)	54 (45–61)	45 (31–61)	0.01
Male (*n*, %)	124 (62%)	38 (63%)	37 (62%)	15 (50%)	31 (52%)	0.46
Body mass index (kg/m^2^)	25.9 (22.7–29.1)	24.4 (20.7–27.7)	24.8 (22.2–27.5)	28.7 (24.8–34.5)	25.2 (22.5–28.7)	0.001
Patients without CLD (*n*, %)	126 (63%)	30 (50%)	32 (53%)	0 (0%)	25 (42%)	< 0.001
Volunteers (*n*, %)	0 (0%)	0 (0%)	0 (0%)	0 (0%)	25 (42%)	< 0.001
Patients with CLD (*n*, %)	74 (37%)	30 (50%)	28 (47%)	30 (100%)	35 (58%)	< 0.001
PSC (*n*, %)	1 (1%)	30 (100%)	28 (100%)	1 (3%)	1 (2%)	< 0.001
MASLD (*n*, %)	29 (15%)	0 (0%)	0 (0%)	18 (60%)	14 (23%)	< 0.001
ARLD (*n*, %)	27 (14%)	0 (0%)	0 (0%)	4 (13%)	4 (7%)	< 0.001
Viral hepatitis (*n*, %)	12 (6%)	0 (0%)	0 (0%)	2 (7%)	5 (8%)	0.03
Fibrosis F0-F2 (*n*, %)	16 (8%)	24 (80%)	22 (79%)	22 (73%)	19 (32%)	< 0.001
Fibrosis F3-F4 (*n*, %)	58 (29%)	7 (23%)	5 (18%)	8 (27%)	16 (27%)	< 0.001
Liver volume, cm^3^	1,482 ± 385	1,026 ± 632	1,070 ± 622	1,723 ± 429	1,516 ± 446	< 0.001
Liver segments I-III volume, cm^3^	334 ± 146	235 ± 141	245 ± 140	350 ± 125	336 ± 125	< 0.001
Liver segments IV-VIII volume, cm^3^	1,148 ± 316	791 ± 536	825 ± 515	1,373 ± 354	1,179 ± 381	< 0.001

Age and body mass index are presented as median with interquartile range (25–75%). Other values are presented as *n* (%) or mean ± SD. *p*-values were calculated using the Kruskal–Wallis test or χ^2^-test as appropriate

*ARLD* Alcohol-related liver disease, *CLD* Chronic liver disease, *MASLD* Metabolic dysfunction-associated steatotic liver disease, *PSC* Primary sclerosing cholangitis

### Optimization of the sequence-adaptive mpMRI quantification

Multiple combinations of hyperparameters were evaluated and compared, as presented in Appendix E3 (Supplementary Material). The best configuration included liver segmental values based on minimally 50 analyzed voxels, removal of voxel outliers by total liver median ± 3 * median absolute deviation, and high entropy detection. The final algorithm is formally defined in Appendix E4 (Supplementary Material).

### Comparison of automated and manual quantification

The ICC per Couinaud liver segment ROI mean and total liver ROI mean are presented in Table 3. The reliability of the segmental analysis between the algorithm and manual assessment in the internal testing dataset was excellent, with a mean ± standard deviation ICC of 0.93 ± 0.07 for noncontrast T1 relaxation times, 0.99 ± 0.00 for post-contrast T1 relaxation times, and 0.96 ± 0.02 for T2 relaxation times (*p* < 0.001). The reliability of the segmental analysis of the segmental external testing dataset was excellent, with a mean ± standard deviation ICC of 0.96 ± 0.02 for noncontrast T1 relaxation times, 0.97 ± 0.02 for post-contrast T1 relaxation times, and 0.90 ± 0.04 for T2 relaxation times (*p* < 0.001). The reliability of the total liver external testing dataset was excellent as well, with a mean ICC between 0.94 and 0.99 for all relaxation time mapping sequences (*p* < 0.001).

**Table 3:** Inter-rater reliability between automated and manual T1 and T2 relaxation time quantification

	Noncontrast T1		Contrast-enhanced T1		T2	
Internal testing, segmental	ICC	*p*-value	ICC	*p*-value	ICC	*p*-value
Segment I	0.97 [0.95, 0.98]	< 0.001	> 0.99 [0.99, 1.0]	< 0.001	0.98 [0.96, 0.99]	< 0.001
Segment II	0.82 [0.71, 0.89]	< 0.001	0.99 [0.99, 1.0]	< 0.001	0.92 [0.87, 0.95]	< 0.001
Segment III	0.79 [0.65, 0.88]	< 0.001	> 0.99 [0.99, 1.0]	< 0.001	0.93 [0.88, 0.96]	< 0.001
Segment IVa	0.97 [0.94, 0.98]	< 0.001	> 0.99 [0.99, 1.0]	< 0.001	0.96 [0.94, 0.98]	< 0.001
Segment IVb	0.96 [0.93, 0.98]	< 0.001	0.99 [0.97, 0.99]	< 0.001	0.98 [0.96, 0.99]	< 0.001
Segment V	0.98 [0.97, 0.99]	< 0.001	0.99 [0.97, 0.99]	< 0.001	0.93 [0.88, 0.96]	< 0.001
Segment VI	0.96 [0.93, 0.98]	< 0.001	0.99 [0.98, 1.0]	< 0.001	0.97 [0.94, 0.98]	< 0.001
Segment VII	0.98 [0.96, 0.99]	< 0.001	> 0.99 [0.99, 1.0]	< 0.001	0.97 [0.94, 0.98]	< 0.001
Segment VIII	0.95 [0.92, 0.97]	< 0.001	> 0.99 [0.99, 1.0]	< 0.001	0.97 [0.95, 0.98]	< 0.001
All segments	0.93 ± 0.07 [0.88, 0.98]		0.99 ± 0.00 [0.99, 1.0]		0.96 ± 0.02 [0.94, 0.97]	
**External testing, segmental**	**ICC**	***p*-value**	**ICC**	***p*-value**	**ICC**	***p*-value**
Segment I	0.97 [0.92, 0.99]	< 0.001	0.97 [0.95, 0.99]	< 0.001	0.90 [0.80, 0.95]	< 0.001
Segment II	0.97 [0.94, 0.98]	< 0.001	0.97 [0.93, 0.98]	< 0.001	0.85 [0.71, 0.92]	< 0.001
Segment III	0.96 [0.91, 0.98]	< 0.001	0.97 [0.93, 0.99]	< 0.001	0.92 [0.82, 0.96]	< 0.001
Segment IVa	0.97 [0.95, 0.99]	< 0.001	0.96 [0.92, 0.98]	< 0.001	0.85 [0.71, 0.92]	< 0.001
Segment IVb	0.93 [0.84, 0.97]	< 0.001	0.96 [0.91, 0.98]	< 0.001	0.90 [0.79, 0.95]	< 0.001
Segment V	0.98 [0.96, 0.99]	< 0.001	0.98 [0.95, 0.99]	< 0.001	0.96 [0.91, 0.98]	< 0.001
Segment VI	0.97 [0.93, 0.98]	< 0.001	0.93 [0.86, 0.97]	< 0.001	0.95 [0.90, 0.98]	< 0.001
Segment VII	0.93 [0.85, 0.96]	< 0.001	0.93 [0.86, 0.97]	< 0.001	0.88 [0.77, 0.94]	< 0.001
Segment VIII	0.94 [0.88, 0.97]	< 0.001	0.94 [0.88, 0.97]	< 0.001	0.88 [0.76, 0.94]	< 0.001
All segments	0.96 ± 0.02 [0.94, 0.97]		0.97 ± 0.02 [0.95, 0.97]		0.90 ± 0.04 [0.87, 0.92]	
**External testing, total liver**	**ICC**	***p*-value**	**ICC**	***p*-value**	**ICC**	***p*-value**
Whole liver	0.99 [0.98, 0.99]	< 0.001	0.98 [0.96, 0.98]	< 0.001	0.94 [0.91, 0.96]	< 0.001

Results are presented as ICC with 95% confidence interval and corresponding *p*-value

*ICC* Intraclass correlation coefficient, *CI* Confidence interval

### Disagreement between automated and manual quantification

**Fig. 3:**
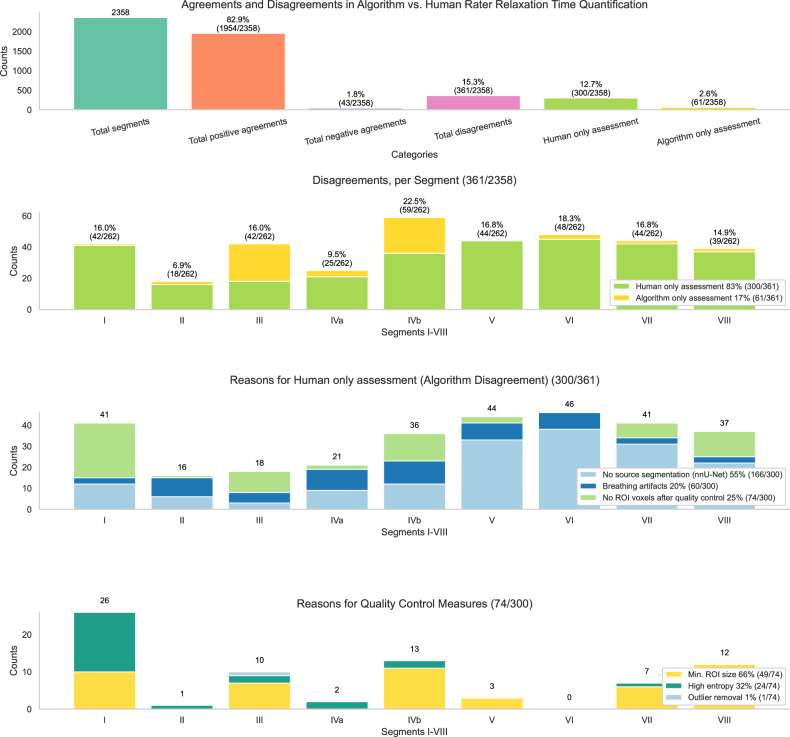
Disagreement analysis between the manual and automated mpMRI quantification. The top plot shows the distribution of positive and negative agreements, as well as disagreement categories. The second plot details reasons for disagreements per segment, while the third plot highlights cases where only the human reader labeled a liver segment, but the algorithm did not. The bottom plot unbundles the reasons for cases where the algorithm initially assessed a liver segment but then omitted the measurement due to quality control measures

As illustrated in Fig. 3, among a total of 2,358 measurable liver segments, agreement between automated and manual quantification of segmental relaxation time measurements was high in all testing datasets, with 1,954/2,358 positive agreements to measure a liver segment (82.9%), followed by another 43/2,358 negative agreements not to measure a liver segment (1.8%). Disagreements (15.3%) mostly occurred when the human reader chose to perform a manual ROI measurement on a liver segment, but the algorithm did not (12.7%, 300/2,358). The main reasons were the nnU-Net segmentation algorithm did not delineate a segment in the first step (55%, 166/300) or when the patient movement between MRI sequence acquisitions caused relaxation time maps to be misaligned with the source segmentation of the T1-weighted VIBE Dixon acquisitions (breathing artifacts; 20%, 60/300). The third reason for disagreement arose when no voxels remained after completing the second step, the co-registration and quality control measures (25%, 74/300). The issues included fewer than 50 voxels available from a liver segment (minimum ROI size), excessive noise in the liver segment (high entropy), or too few voxels remaining after voxel outlier removal to proceed with the measurement. In contrast, the human reader was free to adapt liver segmental borders and was more likely to ensure a complete assessment by allocating at least 50 voxels to all nine liver segments.

### Accuracy of measured T1 and T2 relaxation times

**Fig. 4:**
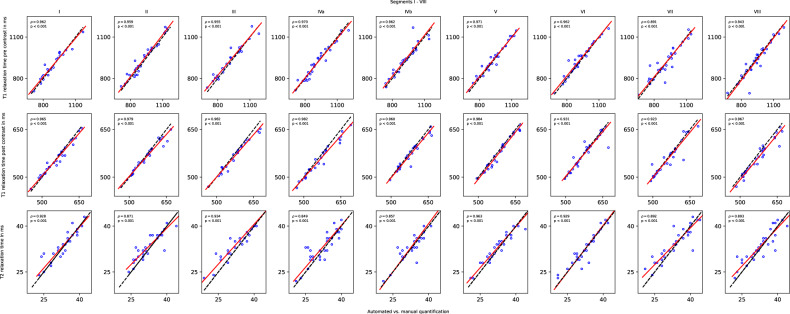
Scatterplots show concordance between automated and manual parametric T1 and T2 relaxation time quantification for the external testing dataset. Perfect concordance is represented by a 45-degree line (black line), and the observed concordance is represented by the Deming regression slope (red line). All pairwise comparisons show a significant Spearman correlation (*p* < 0.001)

**Fig. 5:**
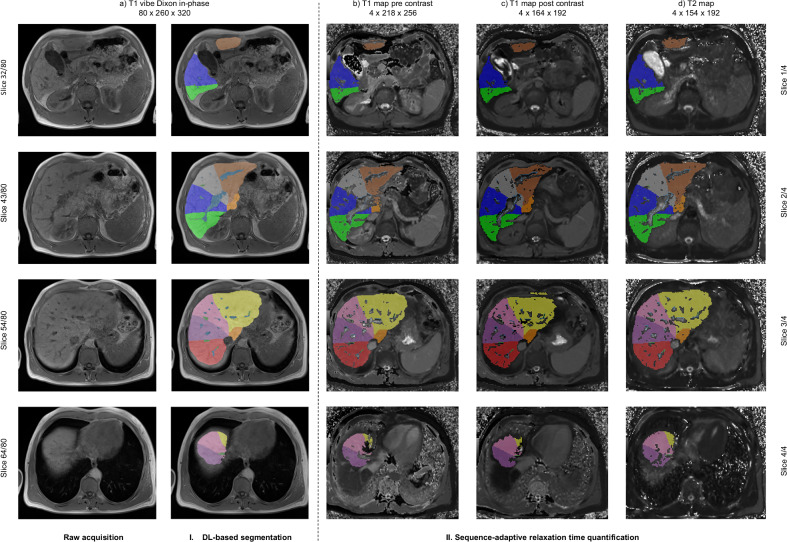
Example of automated T1 and T2 relaxation time quantification. A fully automated sequence-adaptive T1 and T2 relaxation time quantification in a 42-year-old male patient with primary sclerosing cholangitis that was previously unseen by the algorithm is shown. Automated liver segmental labeling by the DL algorithm on noncontrast T1 VIBE Dixon in-phase images is shown in **a**, by excluding liver portal and hepatic veins. Sequence-adaptive quantification transformed the segmental labels to different parametric maps, including (**b**) T1 map pre-contrast, (**c**) T1 map post-contrast and (**d**) T2 map. The maps consisted of four axial slices. Each slice was mapped to its corresponding Euclidean nearest-neighbor slice in the T1 VIBE Dixon in-phase sequence using the affine matrices of both sequences. In this example, T1/T2 map slice 1/4 to in-phase slice 32/80, T1/T2 map slice 2/4 to in-phase slice 43/80, T1/T2 map slice 3/4 to in-phase slice 54/80, and T1/T2 map slice 4/4 to in-phase slice 64/80

**Fig. 6:**
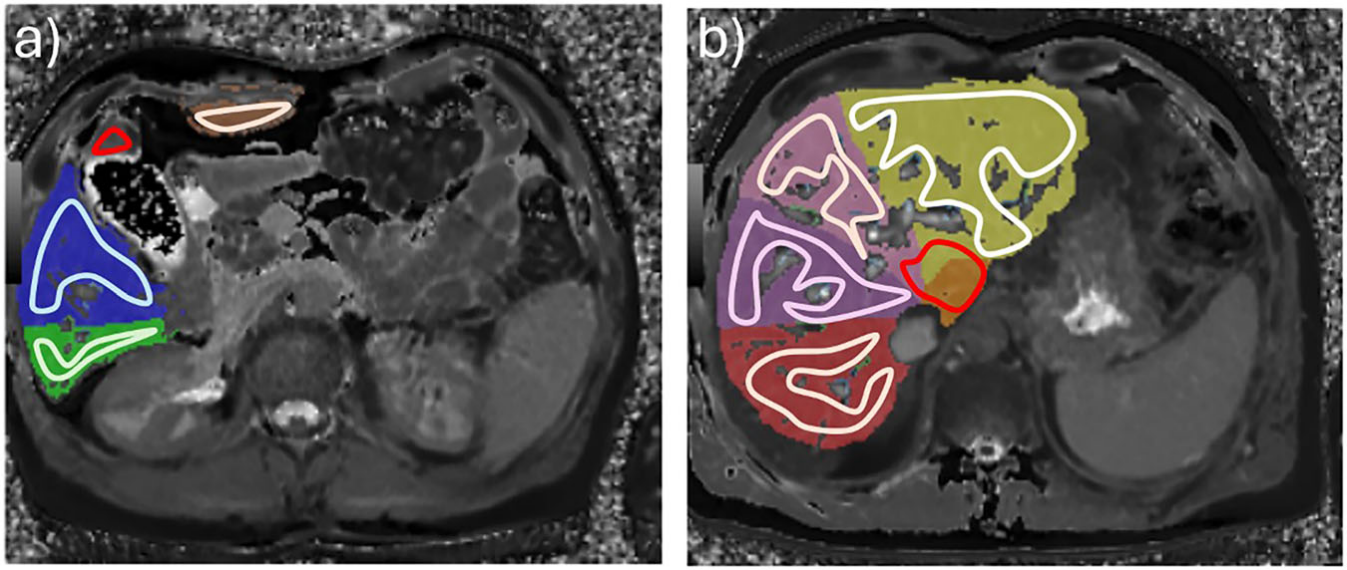
Comparison of automated and manual relaxation time quantification: an example using two representative slices of T1 mapping in one patient. The two regions of interest (ROI) in red highlight (**a**) a part of the liver segment IVb, measured by the human reader but neglected by the algorithm, and (**b**) liver segment I, which was measured larger by the human reader than by the algorithm

Very strong correlation of segmental relaxation time measurement was observed, ranging from $\rho$ = 0.85 (*p* < 0.001) for T2 relaxation time in liver segment IVa to $\rho$ > 0.99 (*p* < 0.001) for contrast-enhanced T1 relaxation time in segment I. For a more detailed analysis, Fig. 4 shows scatterplots with Deming regression of all sequence- and segment-wise comparisons of the different relaxation time measurements between the algorithm and manual assessment. The scatterplots for the internal testing dataset are shown in Fig. e2. As an example, a fully automated liver segmental assessment is illustrated in a patient with primary sclerosing cholangitis in Fig. 5. The resulting mean T1 and T2 relaxation time measurements of the liver were highly comparable. The resulting median of the differences between automated and manual relaxation time measurements among all testing populations and liver segments were 1.8 ms for pre-contrast T1 (median 835 *versus* 842 ms), 2.0 ms for post-contrast T1 (median 518 *versus* 519 ms), and 0.3 ms for T2 (median 37 *versus* 37 ms; Table 4). For illustration, automated and manual measurements are shown in Fig. 6 in an example case with large agreement, but also some segmental disagreement between the algorithm and the human reader. Complete results for the automated and manual liver segment ROI means are provided in Table e1 (Supplementary Material).

**Table 4:** Comparison between T1 and T2 map total liver ROI median as quantified by the algorithm and the human reader over all testing datasets

	Automated median, ms	Manual median, ms	Median of differences	*p-*value
T1 relaxation time pre-contrast	835 [810, 863]	842 [805, 866]	1.8	0.883
T1 relaxation time post-contrast	518 [496, 531]	519 [500, 535]	-2.0	< 0.001
T2 relaxation time	37 [35, 38]	37 [35, 38]	0.3	0.002

Values are median and 95% confidence interval over all testing datasets. *p-*values were calculated using paired Wilcoxon test (*n* = internal testing + external testing as total liver = 150 patients)

### ROI quantification time of the algorithm

Complete liver segmental parenchyma and vessel segmentation in one test case was performed in 70 s using an NVIDIA GeForce RTX 3090 GPU, an AMD EPYC 7302 16-Core Processor CPU, and an IBM Spectrum Scale-based file system. The automated segmental and total liver T1 and T2 relaxation time quantification took an additional 16 seconds per patient and mpMRI sequence. For comparison, a manual segmental T1 and T2 relaxation time quantification without liver segmental volume quantification took around 4 min per mpMRI sequence. In case of a complete segmental quantification of 3 parametric MRI sequences (for example, noncontrast T1, contrast-enhanced T1, and T2 relaxation times), the total time with the algorithm was less than 2 min, as compared to 12 min with manual segmentation.

## Discussion

This study demonstrates that deep learning-based sequence-adaptive segmental and total liver quantification is feasible on mpMRI. Fully automated segmental and total liver analysis quantification was highly accurate on unseen mpMRI sequences not specifically trained for by the algorithm. This was achieved through a novel two-step approach of liver segmental parenchyma annotation on a standard noncontrast T1-weighted VIBE Dixon sequence, followed by co-registration to parametric mpMRI sequences. The approach was validated with three different T1 and T2 mapping sequences, which varied in slice thickness from the T1-weighted VIBE Dixon source segmentation and were acquired at different time points. Based on our findings, any mpMRI sequence may be automatically quantified based on one single T1-weighted VIBE Dixon source segmentation, provided it is acquired within the same mpMRI exam. Further investigations are warranted to replicate these results with mpMRI sequences at other, external centers, and with additional mpMRI sequences, such as PDFF, IVIM, or magnetic resonance elastography measurements. The proposed algorithm could enable the automation of ROI quantification across a dynamically determined set of sequences as part of routine clinical workflows. It could provide a comprehensive quantitative liver phenotype dashboard, streamlining tasks such as evaluating liver fibrosis, assessing treatment response, or monitoring disease progression, thereby allowing radiologists to make faster, more robust, and personalized decisions.

The excellent reliability between automated and manual quantification is underlined by the high accuracy of the resulting T1 and T2 relaxation times. The resulting values were not only comparable with median of the differences, but also comparable with previously published manual assessment studies. The average noncontrast T1 was reported as 802 ± 55 ms for healthy females, 759 ± 69 ms for healthy males [29], and 783 ± 88 ms in healthy children [30]. Other studies assessed T1 relaxation time in different populations with and without CLD, including 767 ± 82 ms in patients without CLD [31], 781 ± 90 ms in patients with liver fibrosis [1], and 991 (891–1,091) ms in patients with decompensated portal hypertension [32]. The average T2 time was comparable with similar studies as well, including 36 ± 4 ms in 147 participants without liver disease [33], 34 ± 4 ms in healthy volunteers [34], and 39 ± 7 ms in a mixed cohort of patients with and without CLD [35].

To our knowledge, this is the first approach to automatically transfer segmentation masks to arbitrary mpMRI sequences. Some studies trained neural networks directly on T1 or T2 maps for automated measurement [19, 20] and other modalities [36, 37]. Lefebvre et al [21] used mpMRI data to train a model for disease prediction without generating segmental mpMRI data. Huo et al [38] proposed a method for automatic liver attenuation ROI-based measurement on CT. These approaches, however, do not scale to new sequences and require re-training for each sequence. In contrast, the proposed algorithm annotates and quantifies liver segments from unseen mpMRI data without specific training. Another advantage is its speed. Manually, the average time for a complete segmental analysis is around 4 min per mpMRI sequence at our institution. The automated quantification completes the task in 86 seconds per sequence, saving significant time and effort, especially when multiple mpMR sequences are combined. Furthermore, the algorithm is easily extendable to compute additional metrics, such as volume, within a single computation.

Despite excellent reliability in this study, small differences occurred between automated and manual assessments. The algorithm measured more restrictively, omitting 300 out of 2,358 segmental measurements. The main reasons were that the parametric T1 and T2 maps were acquired on 4 single slices, and the algorithm did not find a liver segment on the corresponding T1-weighted VIBE Dixon sequence segmentation. Other reasons included patient movement between acquisitions and calculated ROIs that were too small or had high entropy. Apparently, the human reader tended to allocate measurements to all nine liver segments, even if those segments were not adequately covered. In addition, humans were not able to omit potentially erroneous measurements from high-entropy regions. Arguably, high entropy and movement artifacts are the two most significant factors contributing to discrepancies between human and algorithmic measurements, due to their nonlinear and hard-to-formalize nature. These phenomena pose distinct challenges that the algorithm addresses more restrictively, while human readers adapt more flexibly but at the cost of potential errors. Despite its restrictiveness, the algorithm’s resulting parameters were systematically measured and showed very strong comparability to manual measurements. A high reproducibility and robustness of the measurements are mandatory when combining different mpMRI sequences, such as T1 relaxation time mapping before and after contrast medium administration for ECV calculation [16], or the combination of T1 mapping of the fat and water fraction with and without PDFF assessment [39]. Therefore, clinical validation trials should now compare automated to manual assessment of mpMRI data in patients with different degrees of liver fibrosis, steatosis and inflammation. Thanks to the sequence-adaptive design of the proposed two-step approach, such trials will be possible. Moreover, the algorithm suits the continuously evolving landscape of present and future mpMR sequences.

This study had limitations. First, this was a single-center study, and all external testing MRI scans were performed on 3-T scanners from one manufacturer, while optimization and internal testing were performed on both 1.5-T and 3-T systems. Therefore, external validation on 1.5-T systems should be performed in subsequent analyses. Another limitation is that the datasets did not include focal liver lesions or postoperative livers. A third limitation is that the algorithm relies on the T1-weighted VIBE Dixon and mpMRI sequences being acquired during the same exam, with no patient movement between sequences for consistent affine transformations. Moreover, the study did not include a sex- and age-specific analysis and did not analyze the performance of the algorithm for characterization of chronic liver disease, which should be performed in a subsequent study. Finally, even if the robustness of the analysis has been shown in different datasets with different liver volumes, external validation is warranted for patients with different liver volumes from different sites.

In conclusion, deep learning sequence-adaptive quantification of liver mpMRI was highly effective across different patient populations based on total and segmental T1 and T2 relaxation map with excellent reliability. The proposed two-step approach allows this assessment to be extended to any mpMRI sequence, thereby enhancing individual liver phenotyping. Future research should prioritize multicenter studies to validate the algorithm’s generalizability and reliability for clinical adoption and assess its performance on additional mpMRI sequences.

## Supplementary information

**Additional file 1:** **Table e1.** Comparison between mean segmental T1 and T2 relaxation times as quantified by the algorithm and the human reader in segmental testing datasets. **Figure e1. Top-20 parameter configurations of the sequence-adaptive mpMRI quantification algorithm on the optimization dataset**. The *f*-score represents the configuration performance according to the objective function *f*. The Mean ICC Value represents the mean ICC and standard deviation over all segments and all available mapping sequences per configuration. The Total ROI Measurements refer to the number of measurements the algorithm performed under the given configuration. The percentage represents the number of measurements performed by the algorithm compared to the human reader. The x-axis shows each configuration consisting of the proposed algorithm components, presented as follows: <erosion>_<SS-enabled>_<SS-neighborhood>_<low entropy cut-off>_<entropy threshold low>_<minimum ROI size>, where SS means NCC-based source slice subselection heuristic. **Figure e2. Scatterplots show concordance between automated and manual parametric T1 and T2 relaxation time quantification for the internal testing dataset**. Perfect concordance is represented by a 45-degree line (black line), and the observed concordance is represented by the Deming regression slope (red line). All pairwise comparisons show a significant Spearman correlation (*p* < 0.001). Best viewed in screen.

## Data Availability

The datasets generated and/or analyzed during the current study are available from the corresponding author on reasonable request.

## Abbreviations

CLD: Chronic liver disease
DL: Deep learning
ICC: Intraclass correlation coefficient
IVIM: Intra-voxel incoherent motion
mpMRI: Multiparameric magnetic resonance imaging
PDFF: Proton density fat fraction
ROI: Region of interest
VIBE: Volumetric interpolated breath-hold examination
