# Supplementary material for: Automated quantification of T1 and T2 relaxation times in liver mpMRI using deep learning: a sequence-adaptive approach
Source: Eur Radiol Exp. 2025 Jun 14;9:58. doi: 10.1186/s41747-025-00596-9 (PMC12167186; doi:10.1186/s41747-025-00596-9)
Supplement: Supplementary file 1 — Additional file 1: Table e1. Comparison between mean segmental T1 and T2 relaxation times as quantified by the algorithm and the human reader in segmental testing datasets. Figure e1. Top-20 parameter configurations of the sequence-adaptive mpMRI quantification algorithm on the optimization dataset. The f-score represents the configuration performance according to the objective function f. The Mean ICC Value represents the mean ICC and standard deviation over all segments and all available mapping sequences per configuration. The Total ROI Measurements refer to the number of measurements the algorithm performed under the given configuration. The percentage represents the number of measurements performed by the algorithm compared to the human reader. The x-axis shows each configuration consisting of the proposed algorithm components, presented as follows: _____, where SS means NCC-based source slice subselection heuristic. Figure e2. Scatterplots show concordance between automated and manual parametric T1 and T2 relaxation time quantification for the internal testing dataset. Perfect concordance is represented by a 45-degree line (black line), and the observed concordance is represented by the Deming regression slope (red line). All pairwise comparisons show a significant Spearman correlation (p < 0.001). Best viewed in screen. [file 41747_2025_596_MOESM1_ESM.pdf]

Automated quantification of T1 and T2 relaxation times in liver mpMRI using deep learning: a sequence-adaptive approach

ELECTRONIC SUPPLEMENTARY MATERIAL

Appendix E1

Sequence-adaptive mpMRI quantification algorithm components

Subsequently, we detail the proposed algorithm with all devised components aimed at optimal mpMRI region-of-interest (ROI) quantification. We use *source* sequence to refer to the T1 vibe Dixon in-phase acquisition and *target* sequence to refer to any of the mapping sequences, including T1 map pre contrast, T1 map post contrast, and T2 map. Since the target sequence has a lower resolution than the source sequence (Table 1), transformation conflicts can occur where multiple source labels map to the same target voxel. These conflicts are resolved by Euclidean nearest neighbor. Subsequently, the additional proposed algorithm components are described: 1) ROI erosion: Due to partial volume effects, erosion is performed on the target ROI to remove potentially ambiguous voxels at the boundary; 2) Minimum ROI size per slice (for robustness): the algorithm should omit a measurement if the size of the target ROI is below a threshold; 3) Removal of voxel outliers: to make the ROI mean computation more robust against image artifacts, statistical voxel outliers are removed. The median and the median absolute deviation (MAD) of the 3D total liver parenchyma ROI on the target map is computed. The median and the MAD are then used to remove any voxels within the ROI that are outside the range defined by median ± 3 * MAD; 4) High entropy detection: the algorithm should omit a measurement if the amount of entropy inside the target ROI is too high, i.e., is above a threshold; 5) Source slice sub-selection: the axial slice mapping between source and target sequence occurs through the affine matrix. The stringently obtained source slice might not be correct in the case of patient breathing. Normalized cross-correlation (NCC) based source slice sub-selection scans neighboring source slices to find higher ROI-NCC with the corresponding target sequence and selects accordingly.

Following, we describe some of the devised components in more detail.

ROI erosion

Erosion is a morphological operation that reduces the borders of a foreground object. In MRI, eroding the ROI along its borders aims to minimize partial volume effects. The erosion procedure is defined by the size of the 2D kernel and the number of erosion iterations.

High entropy detection

In MRI axial slices, high entropy areas within the abdomen can occur due to several factors, including motion artifacts, complex anatomical structures, partial volume effects, chemical shift artifacts, and heterogeneous pathologies. Conversely, an axial slice of a mapping sequence with good acquisition quality typically exhibits comparably low entropy within the liver area. To mitigate the risk of measuring a noisy ROI, we calculate the entropy within the ROI area per slice and ensure the fraction of low entropy values within the ROI is sufficiently large (parameter τ). An entropy value is considered low if it falls below a specified threshold (parameter $threshold_{low}$). If these constraints are not met, the algorithm omits the measurement of the ROI on the given axial slice.

NCC-based source slice sub-selection heuristic

We propose a heuristic component to determine the best source sequence slice in the presence of breathing artifacts in either the source or target sequence. The algorithm determines for each axial target mapping slice the corresponding source in-phase slice by using the respective affine matrix. However, due to breathing artifacts, the mathematically obtained source sequence slice might not represent the semantically correct slice. To address this, we employ normalized cross-correlation (NCC) in the immediate orthogonal neighborhood of the source sequence slice (2-3 slices in both z-directions) as a heuristic to find the source slice that best correlates with the target mapping slice within the ROI.

Appendix E2

Sequence-adaptive mpMRI quantification algorithm optimization

The algorithm required a multi-objective function that optimized two criteria simultaneously: 1) the mean intraclass correlation coefficient (ICC), as it represents a common measure in reliability and agreement analysis between a machine learning-based algorithm and a human reader; and 2) the number of ROI measurements to ensure clinical utility. A trade-off occurs when an increased number of measurements negatively impacts the ICC value, or vice versa. We used ICC(1,1) to compare our algorithm to the gold standard. Consequently, we formulated the multi-objective function as follows: $f = w_1 \cdot ICC_{mean} - w_2 \cdot ICC_{std} + w_3 \cdot |ROIs|_{norm}$, where ICC is the mean and standard deviation over all ROIs, $|ROIs|_{norm}$ is the normalized number of measurements over all ROIs, and w_1, w_2, w_3 the weights for the respective criteria. The optimization was performed separately for each target sequence and type of ROI, i.e. each of the nine Couinaud liver segments. The parameter search space included all the proposed algorithm components as described above. We applied grid search to exhaustively search all combinations of hyperparameter values and find a Pareto optimal algorithm configuration. Depending on the configuration, the algorithm was designed to omit the quantification of a ROI if not all requirements of all components were met, explaining the reason for better interpretability. The algorithm always calculated the ROI mean volumetrically (3D) from all axial slices containing the same ROI.

Appendix E3

Sequence-adaptive mpMRI quantification algorithm optimization results

The top-20 results of the optimization are presented in Figure e1. All configurations used total liver ROI median ± 3 * MAD for voxel outlier removal. The best configuration achieved a mean ICC of 0.9668 ± 0.02 with 1189 measurements out of 1620, 79.7% (1189/1492) of the number of measurements of the manual human reader. It comprised of the following hyperparameters (algorithm components): high entropy detection with τ = 0.05, $threshold_{low}$ = 2.1, no source slice sub-selection, and a minimum ROI size per slice of 50 voxels.

Appendix E4

Sequence-adaptive mpMRI quantification algorithm formal definition

Algorithm 1 shows the final sequence-adaptive automated mpMRI quantification algorithm as employed in the testing phase, using the best configuration obtained from the optimization phase.

Algorithm 1 Sequence-adaptive mpMRI quantification

Require: f_θ trained segmentation network, v_s source sequence, v_t target sequence, r ROI label, $\text{threshold}_{\text{low}}$ low entropy cut-off, τ min low entropy fraction

$\text{seg}_s \leftarrow f_\theta(v_s)$

$\text{roi}_s = \text{seg}_s \cdot \mathbf{1}_{\{\text{seg}_s = r\}}$

$A_s \leftarrow \text{meta}(v_s)_{\text{affine}}$ ▷ extract affine matrix

$A_t \leftarrow \text{meta}(v_t)_{\text{affine}}$

for all k **do** ▷ for each target slice k locate source slice k'

  $\text{roi}_{t,k,\text{cand}} = \emptyset$

  $\text{roi}_{t,k} = \emptyset$

  $\begin{bmatrix} 0 \\ 0 \\ k' \\ 1 \end{bmatrix} \leftarrow A_s^{-1} \cdot A_t \begin{bmatrix} 0 \\ 0 \\ k \\ 1 \end{bmatrix}$

  for all $i', j' \in v_s[k']$ **do** ▷ for each source voxel

    $\begin{bmatrix} i \\ j \\ k \\ 1 \end{bmatrix} \leftarrow A_t^{-1} \cdot A_s \begin{bmatrix} i' \\ j' \\ k' \\ 1 \end{bmatrix}$ ▷ collect ROI label candidates

    $\text{roi}_{t,k,\text{cand}}[i,j] \leftarrow \text{roi}_{t,k,\text{cand}}[i,j] \cup (i, j, k, \text{roi}_s[j', i', k'])$

  end for

  for all $i, j \in \text{roi}_{t,k,\text{cand}}$ **do** ▷ resolve target label conflicts

    $\text{roi}_{t,k}[i,j] \leftarrow \arg\min_{l \in \{l' \mid (x,y,z,l') \in \text{roi}_{t,k,\text{cand}}[i,j]\}} \sqrt{(x-j)^2 + (y-i)^2 + (z-k)^2}$

  end for

  reject quantification for k if $size(\text{roi}_{t,k}) <$ min. ROI size

  $\text{E}_{roi_{t,k}} \leftarrow [H(i, j, v_t[k]) \mid \forall (i,j) \text{ s.t. } \text{roi}_{t,k}[i,j] = r]$ ▷ compute entropy

  $\text{fraction}_{\text{low entropy}} = \frac{\sum_{i=1}^{card(\text{E}_{\text{roi}_{t,k}})} \mathbf{1}(\text{E}_{\text{roi}_{t,k}}(i) < \text{threshold}_{\text{low}})}{card(\text{E}_{\text{roi}_{t,k}})}$

  reject quantification for k if $\text{fraction}_{\text{low entropy}} < \tau$

  $\text{stats}[k]_{roi_t} \leftarrow$ collect statistics from $\text{roi}_{t,k}, v_t[k]$

end for

$\text{vox}_{\text{roi}_t} \leftarrow voxels(\text{stats}_{\text{roi}_t})$ ▷ Retrieve all voxels in roi_t

$3\text{mad} = 3 * mdn(|\text{vo}_i - mdn(\text{vox}_{\text{roi}_t})| \quad \text{for} \quad \text{vo}_i \in \text{vox}_{roi_t})$

$\text{vox}_{t,\text{inliers}} = \{\text{v} \in \text{vox}_{\text{roi}_t} \mid mdn(\text{vox}_{\text{roi}_t}) - 3\text{mad} \leq \text{v} \leq mdn(\text{vox}_{\text{roi}_t}) + 3\text{mad}\}$

return $\mu(\text{vox}_{t,\text{inliers}})$

Table e1. Comparison between mean segmental T1 and T2 relaxation times as quantified by the algorithm and the human reader in segmental testing datasets.

**T1 relaxation time pre contrast**	**automated median, msec**	**manual median, msec**	**median of differences**	***p*-value**
Segment I	792 [740, 823]	778 [727, 815]	9	0.01
Segment II	825 [763, 851]	818 [764, 850]	10	0.015
Segment III	825 [778, 867]	803 [751, 860]	8	0.004
Segment IVa	793 [741, 835]	796 [713, 825]	10	0.004
Segment IVb	839 [763, 864]	810 [743, 879]	5	0.037
Segment V	832 [766, 877]	821 [756, 878]	6	0.028
Segment VI	848 [785, 883]	842 [773, 877]	6	0.001
Segment VII	793 [748, 863]	777 [740, 852]	4	0.112
Segment VIII	803 [752, 863]	819 [740, 849]	-1	0.677
**T1 relaxation time post contrast**	**automated median, msec**	**manual median, msec**	**median of differences**	***p*-value**
Segment I	380 [340, 486]	377 [338, 485]	0	0.412
Segment II	375 [342, 483]	379 [344, 485]	-1	0.064
Segment III	372 [333, 470]	373 [335, 472]	-1	0.063
Segment IVa	362 [330, 466]	367 [331, 472]	-1	0.03
Segment IVb	385 [335, 525]	385 [342, 528]	-1	0.006
Segment V	402 [364, 516]	408 [365, 524]	-3	<0.001
Segment VI	411 [357, 525]	411 [354, 529]	-1	0.13
Segment VII	390 [353, 506]	397 [356, 504]	-1.5	0.026
Segment VIII	378 [351, 506]	384 [353, 514]	-4	<0.001
**T2 relaxation time**	**automated median, msec**	**manual median, msec**	**median of differences**	***p*-value**
Segment I	38 [36, 40]	38 [35, 40]	0	0.558
Segment II	37 [36, 40]	38 [36, 41]	1	0.042
Segment III	38 [36, 40]	37 [35, 40]	1	0.003
Segment IVa	38 [36, 41]	37 [36, 42]	0.1	0.239
Segment IVb	37 [36, 42]	38 [35, 42]	0	0.165
Segment V	37 [35, 40]	38 [34, 40]	0	0.654
Segment VI	38 [35, 41]	38 [36, 41]	0	0.488
Segment VII	39 [36, 42]	40 [37, 42]	0	0.252
Segment VIII	37 [35, 41]	38 [35, 39]	1	0.005

Values are median and 95% confidence interval. *P*-values were calculated using paired Wilcoxon test.

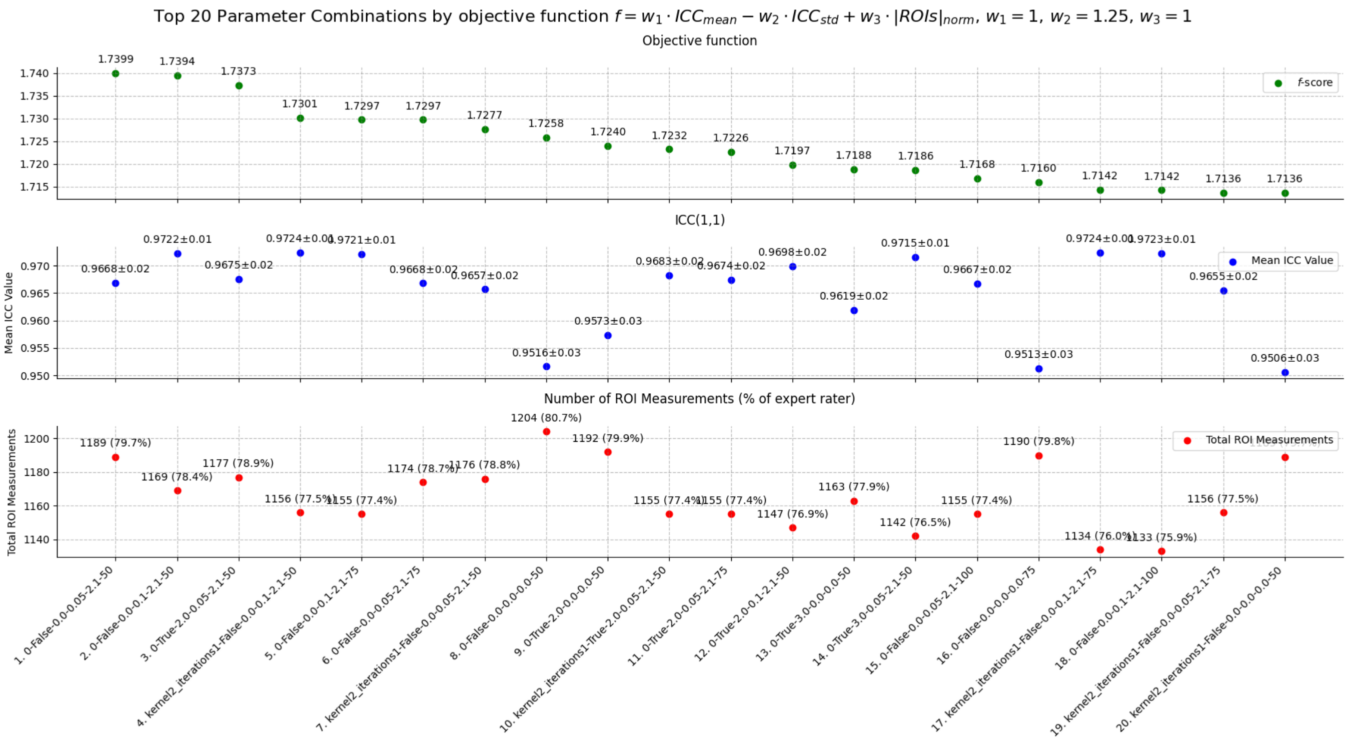

Figure e1: Top-20 parameter configurations of the sequence-adaptive mpMRI quantification algorithm on the optimization dataset. The *f*-score represents the configuration performance according to the objective function f . The Mean ICC Value represents the mean ICC and standard deviation over all segments and all available mapping sequences per configuration. The Total ROI Measurements refer to the number of measurements the algorithm performed under the given configuration. The percentage represents the number of measurements performed by the algorithm compared to the human reader. The x-axis shows each configuration consisting of the proposed algorithm components, presented as follows: <erosion>_<SS-enabled>_<SS-neighborhood>_<low entropy cut-off>_<entropy threshold low>_<minimum ROI size>, where SS means NCC-based source slice subselection heuristic.

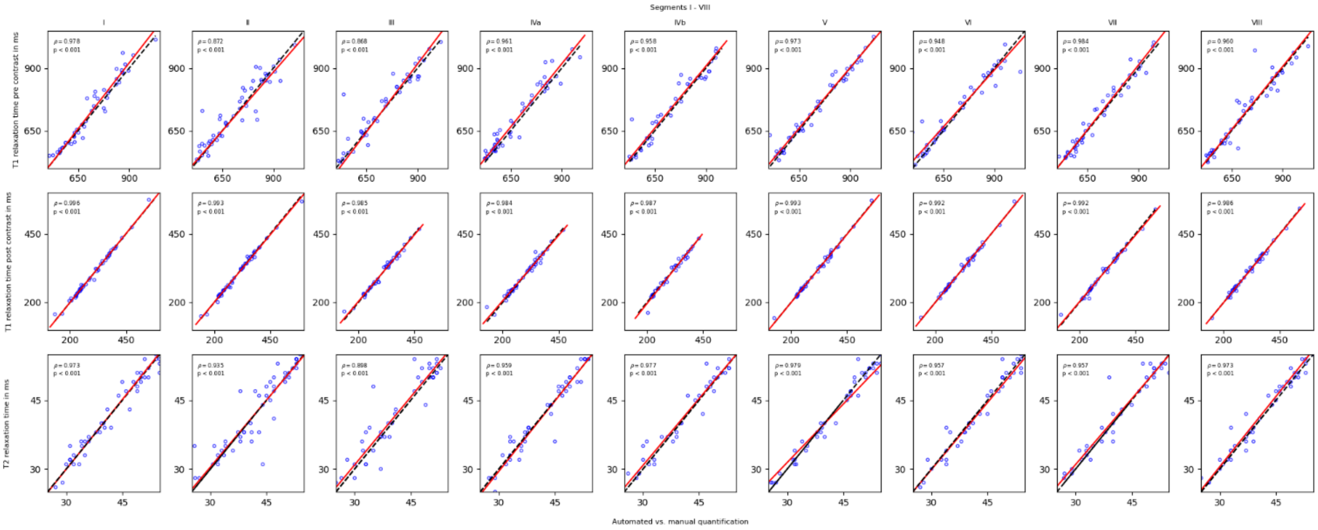

Figure e2: Scatterplots show concordance between automated and manual parametric T1 and T2 relaxation time quantification for the internal testing dataset. Perfect concordance is represented by a 45-degree line (black line), and the observed concordance is represented by the Deming regression slope (red line). All pairwise comparisons show a significant Spearman correlation ($p < 0.001$). Best viewed in screen.
